# Editorial: Studying plant virus-host interactions: elucidating the natural resistance mechanism

**DOI:** 10.3389/fmicb.2024.1500580

**Published:** 2024-10-17

**Authors:** R. Vinoth Kumar, Prabu Gnanasekaran

**Affiliations:** ^1^Department of Biotechnology, Faculty of Science and Humanities, SRM Institute of Science and Technology, Ramapuram, Chennai, Tamil Nadu, India; ^2^Department of Plant Pathology, Washington State University, Pullman, WA, United States

**Keywords:** plant resistance, susceptibility, plant disease management, phytohormones, transcriptomics

Because plant-infecting viruses are intracellular obligate parasites, they rely heavily on the host (plant) machinery to complete each stage of their infection cycle including replication, transcription, pathogenesis, and spread. Such plant-virus interactions include both host and non-host interactions. Host interactions often cause successful infection and induce the pathogenesis of the viruses within the host cells (host susceptibility) (Garcia-Ruiz, 2018). Non-host interactions, on the other hand, are incompatible in nature and usually do not result in viral infection (host resistance) (Kang et al., 2005). In susceptible hosts, viruses masterfully redirect various host cellular machinery by interacting with host factors to complete their infection cycle (Wang, 2015). In contrast, in resistant plants, natural host resistance (defense) mechanisms restrict or limit the viral infection and disease development within the infection site (Baulcombe, 2004; Soosaar et al., 2005; de Ronde et al., 2014). As plant viruses cause significant crop losses across continents (Jones and Naidu, 2019), the identification of such natural resistance-associated host factors is crucial for devising broad-spectrum antiviral strategies against them. The original research articles included in this Research Topic, “*Studying plant virus-host interactions: elucidating the natural resistance mechanism*”, have identified some of the plant or insect proteins that may provide resistance against plant viruses.

An integrative approach to disease resistance would involve investigating the role of different phytohormones in plant-virus interactions and disease development. Guo et al. showed how exogenous magnesium had a significant impact on Potato virus Y (PVY) resistance in tobacco plants. Illumina RNA sequencing has suggested that this magnesium-based anti-PVY infection was achieved by regulating carbohydrate metabolism and transport, nitrogen metabolism, Ca^2+^ signaling and oxidative phosphorylation. This study also demonstrated that NbTPS and NbGBE act as susceptibility factors whereas NbPPases and NbNR induce resistance against PVY infection. Similarly, Li et al. revealed through Illumina sequencing that phytohormones, glycometabolism, phytochrome interacting factor-cryptochrome-R protein module, and MEKK3/MKK9/MPK7-WRKY33-CML/CDPK module are involved in the pathogenicity of Soybean mosaic virus (SMV). Furthermore, Gupta et al. reported on the differential expression of genes related to autophagy, photosynthetic efficiency, and various phytohormones in papaya plants inoculated with a full-length infectious construct of Papaya ringspot virus (PRSV)-HYD. These studies have suggested the employability of multiple different strategies in response to PVY, PRSV-HYD and SMV infections.

Tritrophic interactions between plant viruses, infected host plants and transmitted insect vectors are indispensable for disease development, pathogenicity and virus spread. Several *Thrips palmi* (insect vector)-derived transcripts related to various defense and signaling pathways were modulated to the infection of orthotospoviruses in thrips. Rajesh et al. studied the effect of *T. palmi* tyrosine kinase Btk29A isoform X1 (Btk29A) and collagen alpha-1 (III) chain-like (COL3A1) on the titer and fitness of Groundnut bud necrosis orthotospovirus (GBNV). This study demonstrated that Btk29A promotes GBNV while COL3A1 resists GBNV infection, suggesting that such factors could be used as novel genetic targets for sustainable thrips management.

The scientific evidence presented in this Research Topic has identified novel disease mechanisms or specific targets that can be potentially used in the development of viral resistance and its management. A better understanding of such regulatory networks in plant resistance mechanisms can help us devise effective disease management strategies to combat plant viral diseases.
